# AI-Driven Problem Solving for Cyber-Physical Systems Security: An Assessment Framework

**DOI:** 10.3390/s26175412

**Published:** 2026-08-27

**Authors:** Vikram Kulothungan, Deepti Gupta, Raju Dhakal, Laxima Niure Kandel

**Affiliations:** 1 Capitol Technology University, Laurel, MD 20708, USA; vikramk1986@gmail.com; 2 Subhani Department of Computer Information Systems, Texas A&M University-Central Texas, Killeen, TX 76549, USA; 3 Department of Electrical Engineering and Computer Science, Embry-Riddle Aeronautical University at Daytona Beach, Daytona Beach, FL 32114, USA; dhakalr@my.erau.edu (R.D.); laxima.niurekandel@erau.edu (L.N.K.)

**Keywords:** Cyber-Physical Systems (CPS), Artificial Intelligence (AI), security and privacy

## Abstract

Cyber-Physical Systems (CPS) are using more AI for smart decisions and automation, but also faces new security issues. This study surveys existing CPS security assessment methodologies across healthcare, automotive, energy, and critical infrastructure, identifying their limitations in addressing emerging digital-physical threats. We find that traditional risk assessment and testing approaches, often network-centric and compliance-driven, are insufficient for AI-powered CPS. New vulnerabilities arise from the tight coupling of cyber and physical components, such as adversarial manipulation of sensors that can cause dangerous misbehavior, supply chain attacks on AI models, and the inability to patch critical devices on the fly. We use AI techniques and problem-solving methods to improve the security of CPS. The framework helps detect threats, monitor system activities, and reduce security risks in real time. It also follows important security and privacy standards such as NIST, IEC 62443, ISO 21434, and GDPR. The system continuously checks CPS operations, uses AI tools to find weaknesses, and supports security compliance. We also study real-world CPS attacks, including industrial malware, car hacking, and medical device attacks, to show the importance of the framework. In this research, we present prototype implementation and experimental evaluation along with a case study of protecting a smart manufacturing plant during a ransomware attack using the proposed approach.

## 1. Introduction

Cyber-Physical Systems (CPS) are engineered integrations of computation with physical processes, enabling innovations from smart healthcare devices and connected vehicles to smart grids and other critical infrastructures. In these safety-critical and regulated domains, CPS must adhere to stringent security, safety, and privacy requirements. The growing infusion of Artificial Intelligence (AI) into CPS, which is enabling autonomous decision-making and adaptive control. It has brought unprecedented capabilities and new security challenges [1]. While AI can enhance CPS operations (for example, by improving real-time decision-making and even threat detection), it also creates dual-edged risks: adversaries may exploit vulnerabilities in AI components or use AI tools to craft sophisticated cyber-physical attacks. Recent high-profile incidents underscore what is at stake. Malware can directly cause physical damage through cyber means, as seen with Stuxnet [2], often cited as the first cyber-physical “weaponized” malware that sabotaged industrial equipment. Likewise, remote hacks of connected cars have demonstrated life-threatening scenarios; for instance, the infamous 2015 Jeep Cherokee incident [3] showed that attackers could remotely seize control of a vehicle’s functions, from disabling the engine on a highway to manipulating brakes and steering. These developments clearly motivate a re-examination of how we assess and ensure CPS security in the age of AI.

Traditional security assessment approaches in CPS-intensive industries are struggling to keep pace with these emerging threats. Regulated sectors like energy, manufacturing, automotive, and healthcare have long relied on established standards and practices (e.g., the NIST Cybersecurity Framework for critical infrastructure, IEC 62443 for industrial control systems, ISO 21434 for automotive cybersecurity) to guide risk assessment. Typically, organizations perform upfront risk analyses, follow static compliance checklists, and conduct periodic penetration testing against known attack scenarios. However, CPS environments are highly dynamic, and complex interactions between cyber and physical components can give rise to emergent behaviors that static, point-in-time analyses miss [4]. Moreover, conventional security tools focused on IT networks or even traditional operational technology (OT) environments often prove inadequate for AI-driven CPS. As Gartner analysts observe, “Industry analysis suggests that network-centric or perimeter defenses alone are no longer sufficient for modern CPS [5]”. Key gaps in current methodologies include limited coverage of the integrity of sensors and actuators (the critical bridge between digital and physical realms), a lack of adversarial machine learning testing to harden AI models, and inflexible, human-intensive assessment processes that cannot adapt in real time to new threats. In summary, today’s CPS security paradigms leave dangerous blind spots when faced with AI-augmented attack surfaces and rapidly evolving hybrid threats.

To address these challenges, this paper proposes an AI-driven security assessment framework for CPS that enables continuous, adaptive protection against evolving cyber-physical threats. In essence, we reimagine the security assessment process itself as a problem that can be augmented by AI techniques, drawing inspiration from principles of creative problem solving in AI. The framework incorporates: (i) dynamic threat modeling to continually formulate and update the understanding of risks, (ii) rich knowledge representation of the CPS (for example, via digital twins or formal models of physical and cyber components) to serve as a canvas for analysis, (iii) automated reasoning and analysis using AI (such as intelligent agents that perform vulnerability scanning, anomaly detection, or attack simulation), and (iv) iterative evaluation and learning that continuously improves the security posture. This AI-driven assessment loop is designed to be proactive and adaptive, in contrast to one-off audits—it can autonomously probe for weaknesses (including those specific to AI components like machine learning models), analyze emerging attack patterns, and recommend mitigations in real-time. Importantly, the framework is built to align with existing safety and security standards, ensuring that organizations can integrate these AI-powered methods without deviating from regulatory requirements. In short, the goal is a security assessment approach that is as intelligent and agile as the AI-enabled CPS it protects, providing a higher level of assurance for systems that are increasingly autonomous and interconnected.

The main contributions of this paper are as follows:

- We conduct a structured literature review across multiple CPS domains to evaluate existing security assessment methodologies and identify limitations.
- We analyze emergent CPS vulnerabilities introduced by AI integration (e.g., adversarial inputs, supply-chain risks, runtime constraints).
- We propose an AI-driven security assessment framework for CPS, incorporating dynamic threat modeling, knowledge-based system representation, AI-guided analysis, and continuous evaluation.
- We present the algorithms, implement a prototype, and validate the approach through experimental evaluation.
- We discuss case studies of CPS security incidents to validate the framework’s relevance and extract lessons and also present a case study on a smart manufacturing plant under ransomware attack.

Together, these contributions lay the foundation for a new paradigm of CPS security assessment that is continuous, adaptive, and intelligence-driven. By introducing automation and AI at key steps while remaining aligned with established best practices, the framework aims to significantly improve defenders’ ability to anticipate and counter sophisticated cyber-physical attacks. The remainder of this paper is organized as follows. Section 2 presents the literature review on CPS security assessment. We discuss CPS vulnerabilities in AI-driven systems in Section 3. Section 4 presents methodologies for securing CPS, and proposed AI-driven CPS security assessment is discussed in Section 5. Section 6 presents prototype implementation and experimental evaluation. Section 7 presents the case study for securing a smart manufacturing plant under ransomware attack. Conclusion and future work are discussed in Section 8.

## 2. Literature Review

Across regulated industries, CPS security assessments have evolved to address domain-specific risks and compliance mandates. In industrial and critical infrastructure, such as energy, utilities, manufacturing, frameworks like the NIST Cybersecurity Framework and IEC 62443 [6,7] series are widely adopted. These emphasize inventorying assets, assessing risks, and implementing layered controls for industrial control systems (ICS). Risk assessment typically follows an asset–threat–vulnerability paradigm, often using methodologies like STRIDE [8] or attack trees to evaluate potential attack paths. However, legacy approaches focus heavily on network segmentation, perimeter defenses, and known vulnerability mitigation, reflecting an IT-centric mindset carried into the OT world. In the automotive domain, the new ISO 21434 [9] standard requires a security-by-design process during development, manufacturers must perform Threat Analysis and Risk Assessment (TARA) on vehicular systems and implement security controls throughout the lifecycle. Automotive security assessments often include extensive penetration testing of in-vehicle networks (CAN, Ethernet) and validation of compliance with requirements (e.g., cryptographic strength, secure boot). The famous 2015 Jeep Cherokee hack served as a wake-up call, it demonstrated remote exploitation leading to physical control over steering and brakes [3], prompting automakers to significantly tighten their assessment practices. Healthcare CPS, such as medical devices and hospital IoT systems, follow guidelines from bodies like the FDA and standards (IEC 80001 [10], ISO 14971 [11] for risk management) that increasingly incorporate cybersecurity. For example, manufacturers must conduct security risk assessments on devices like insulin pumps or pacemakers and provide evidence of controls before regulatory approval. This became prominent after real-world exploits showed the potential for patient harm, e.g., in 2019, the FDA recalled certain insulin pump models when researchers showed attackers could alter insulin dosing remotely [12]. Smart grids and transportation have their own regimes. Electric utilities comply with NERC CIP standards [13], which require regular cyber risk assessments and incident response drills for grid CPS, while rail and aviation have emerging standards addressing integrated safety and security risk analysis. The methodology of each domain addresses the baseline cyber threats and safety hazards, yet AI-driven CPS adds new dimensions that these traditional methodologies were not originally designed to cover.

Beyond assessment standards, recent work has advanced secure-by-design methodologies for CPS. Paredes et al. [14] propose a microservice-based design procedure that builds cyberattack detection, isolation, and component-replica tolerance into real-time control applications, validated on an industrial pH-control process. In the energy domain, Ding et al. [15] review cyber-physical coupling models, security assessment, and recovery strategies for integrated energy systems, while Agarwal et al. [16] combine graph neural networks, transformers, and gradient-boosted trees to jointly detect cyberattacks and predict cascading failures in power grids. These approaches inform our framework’s emphasis on design-time modeling, cross-domain coupling, and AI-driven detection.

A growing body of work also examines AI-driven approaches to CPS security. Comprehensive reviews have surveyed the application of digital twins to cybersecurity using AI [17], as well as the range of anomaly-detection strategies developed to detect threats against CPS. These reviews highlight both the promise of AI-based methods and the absence of an integrated assessment framework that unifies them—a gap this paper addresses. Table 1 summarizes how established methodologies fare across these dimensions and positions our proposed framework against them. Recent studies have explored a broad spectrum of trustworthy AI and cybersecurity challenges, including secure LLM deployment, agentic AI resilience, communication-efficient federated learning, privacy-preserving healthcare, malicious URL detection, AI governance and regulation, blockchain-enabled security, and mission-oriented edge intelligence for Cyber-Physical Systems [18,19,20,21,22,23,24,25,26,27,28].

Several common gaps emerge across traditional CPS security assessments.

- First, static and periodic assessments struggle to keep up with CPS that learn or change behavior over time. Many processes assume a relatively fixed system design once certified, a medical device or vehicle model may not undergo reassessment until a major update. AI-driven functions (e.g., a machine learning model for anomaly detection or autonomous control) could evolve (through updated training) or encounter novel inputs that trigger behavior outside initial specifications. Traditional frameworks lack guidance on continuously evaluating such evolving components.
- Second, there is a cyber-physical scope gap, i.e., security reviews often treat cyber (IT network, software) and physical safety separately. Yet in CPS those domains intersect an attack can traverse from cyber to physical (e.g., malware causing machinery malfunctions) or vice versa (physical tampering enabling a cyber compromise) [29]. For instance, many risk assessments in ICS historically ignored the possibility that an attacker might use physical access (like infecting a USB drive) to introduce malware into an air gap system; Stuxnet vividly demonstrated this gap by using USB propagation to reach Iranian nuclear centrifuges [30].
- Third, conventional methods do not address AI-specific threats. Adversarial machine learning, data poisoning, or model evasion attacks are typically absent from threat catalogs. A standard automotive Threat Agent Risk Assessment (TARA) might consider Controller Area Network (CAN) bus fuzzing or ECU firmware tampering, but not an adversarial camera input that causes an AI vision system to misclassify a stop sign, which can be equally dangerous [31].
- Fourth, supply chain and lifecycle risks are growing. CPS relies on complex supply chains for hardware, software, and AI components. Traditional assessments seldom scrutinize the provenance of training data or pre-trained AI models, yet poisoned data or backdoored models can introduce hidden vulnerabilities. Likewise, hardware Trojans or compromised firmware from third-party suppliers pose risks that may evade typical functional testing.
- Finally, a practical gap is the limited use of automation and AI in the assessment process itself. Security assessments remain labor-intensive, reliant on expert knowledge and checklists that can become outdated. This raises concern given the rapid emergence of new CPS attack techniques; it also suggests an opportunity to apply AI to assist in security evaluations. In summary, while regulated domains have robust security frameworks, these need augmentation to handle emergent digital physical threats and the intricacies of AI-driven systems.

**Table 1:** Comparison of CPS security assessment methodologies across key capability dimensions.

Methodology	Continuous (vs. Periodic)	Cyber–Physical Scope	AI/Adversarial ML Coverage	Automation Level	Compliance Mapping
NIST CSF/RMF	Partial	Partial	No	Low	High
IEC 62443 (ICS)	No	Partial	No	Low	High
ISO/SAE 21434 (TARA)	No	Partial	No	Low	High
Penetration testing	No	Partial	Limited	Medium	Low
AI-based IDS/anomaly	Yes	Partial	Partial	High	Low
**Proposed framework**	**Yes**	**Full**	**Yes**	**High**	**High**

## 3. Emergent CPS Vulnerabilities in AI-Driven Systems

Modern CPS faces a threat landscape that blurs the boundary between digital and physical. Vulnerabilities arising from digital–physical interactions can manifest in ways not seen in traditional IT systems. This section discusses key emergent vulnerabilities and security challenges unique to AI-enabled CPS, illustrated by real incidents and research findings.

### 3.1. Adversarial Manipulation of Sensors and Actuators

The growing reliance on AI for perception and control in CPS creates novel attack surfaces that traditional security measures cannot adequately protect. Machine learning models, particularly those used in computer vision applications, have demonstrated vulnerability to carefully crafted adversarial inputs designed to induce specific misclassifications or incorrect responses. Researchers at the University of Washington have demonstrated a particularly concerning attack vector against autonomous vehicles. Their work showed that ordinary black and white stickers applied to road signs could consistently cause computer vision systems to misclassify them, potentially leading to dangerous driving behaviors. In one striking example, researchers attached small stickers to a standard stop sign that caused an AI vision system to misidentify it as a “Speed Limit 45” sign instead [32]. These attacks were effective at various distances (up to 40 feet) and viewing angles, indicating their practicality in real-world scenarios. What makes these attacks particularly insidious is that they bridge the cyber physical divide, the attack vector exists in the physical domain (modified objects in the environment) but exploits vulnerabilities in the digital realm (AI perception systems). Traditional security controls like firewalls and encryption offer no protection against such threats, as they fundamentally exploit the pattern recognition mechanisms inherent to machine learning models rather than traditional software vulnerabilities. Adversarial attacks extend beyond simple misclassification. Researchers have documented numerous attack methodologies, including gradient-based optimization techniques and carefully calculated perturbations that appear innocuous to human observers but dramatically impact AI systems’ outputs. The manufacturing and industrial sectors face particular risks as they increasingly deploy AI-driven systems for quality control, anomaly detection, and process automation, all of which could be manipulated by an attacker with knowledge of the underlying models [33]. Adversarial perturbations are not limited to vision since similar concepts apply to audio (voice command systems) or even actuator control signals.

### 3.2. Cross-Domain Attack Pathways

The integration of cyber and physical components in modern systems has created unprecedented attack vectors where compromise in one domain can have immediate and potentially catastrophic consequences in the other. This blurring of boundaries demands comprehensive security approaches that address vulnerabilities across both realms. The most notorious example of cross-domain attacks remains Stuxnet, discovered in 2010. This sophisticated malware targeted programmable logic controllers (PLCs) in Iran’s nuclear enrichment facilities, causing physical damage to centrifuges while simultaneously manipulating monitoring systems to report normal operation. By bridging digital infiltration with physical sabotage, Stuxnet demonstrated how cyberattacks could cause tangible destruction in highly sensitive industrial environments. Even more alarming was the 2017 Triton malware attack on a Saudi Arabian petrochemical plant. Unlike previous industrial attacks, Triton specifically targeted safety instrumented systems (SIS), the last line of defense designed to prevent catastrophic accidents. By compromising the Triconex safety controllers manufactured by Schneider Electric, the attackers gained the capability to disable critical safety mechanisms. This would have allowed them to trigger dangerous plant conditions while simultaneously preventing the safety systems from initiating emergency shutdowns or other protective measures. The attack was linked to a state-sponsored group, exemplifying the advanced nature of some cross-domain threats [34]. These sophisticated attacks underscore why the National Institute of Standards and Technology (NIST) emphasizes that effective cyber physical security requires eliminating gaps between physical and cybersecurity approaches [35]. Organizations must conduct comprehensive risk assessments that consider multi-modal attack scenarios and secure both cyber and physical entry points with equal rigor. This holistic approach is essential as the attack surface continues to expand through increased connectivity and automation in critical infrastructure.

### 3.3. Autonomous Decision-Making Errors and Emergent Behaviors

The increasing autonomy and complexity of AI-driven CPS introduce unique challenges related to emergent behaviors, system responses that were not explicitly programmed but arise from the interaction of components and algorithms under unforeseen conditions. These emergent behaviors can manifest as safety or security vulnerabilities that traditional testing methodologies may fail to identify. As systems become more autonomous and incorporate machine learning components, they inherently develop capabilities to operate in scenarios that designers could not fully anticipate during development. This autonomy creates a fundamental gap in security assurance, i.e., that if all possible operational states cannot be predicted at design time, they cannot all be verified as safe before deployment [4]. The unpredictability increases exponentially in collaborative systems where multiple autonomous agents interact, such as drone swarms or multi-robot manufacturing cells. Rare combinations of inputs or environmental conditions can trigger edge-case behaviors in AI systems. Although such scenarios might occur only once in millions of operations, adversaries who discover these weaknesses could deliberately induce them. For example, specially crafted inputs might confuse an AI controller into entering an unsafe state or making decisions that compromise security or safety. The traditional approach of enumerating and testing all possible failure modes becomes mathematically impossible as system complexity increases. Research into protective monitoring frameworks offers promising approaches to mitigate these risks. Advanced monitoring systems can create a “protective shell” around autonomous CPS, continuously evaluating behavior against safety parameters and initiating countermeasures when abnormal patterns are detected [4]. This runtime verification approach complements traditional design-phase security analysis, providing an additional layer of protection against emergent vulnerabilities that could not be anticipated during development.

### 3.4. Supply Chain and Update Insecurities

The extended lifecycle of many CPS creates significant security challenges related to software maintenance and component integrity. Unlike consumer devices with frequent replacement cycles, industrial control systems, medical devices, and infrastructure components often remain in service for decades with minimal opportunities for downtime or updates. This long operational lifespan makes CPS particularly vulnerable to supply chain attacks and update-related security issues. Supply chain attacks against CPS have risen sharply in recent years, with the manufacturing sector among the most exposed owing to its reliance on automation and digitized processes and the value of its intellectual property [36].

Addressing these vulnerabilities requires a comprehensive approach to supply chain security. Organizations must implement robust third-party risk assessments, strengthen vendor oversight procedures, and maintain continuous security monitoring throughout the supply chain ecosystem [37]. Industry experts emphasize that effective security requires balancing operational efficiency with strong cybersecurity protocols, implementing risk mitigation strategies like network segmentation and strict access controls. Looking forward, mandatory Software Bills of Materials (SBOMs), increased regulatory scrutiny, and the integration of AI-powered security monitoring are expected to play central roles in strengthening industrial supply chain security [38]. These measures will provide greater transparency into the components and dependencies within critical systems, enabling more effective vulnerability management and incident response across complex cyber-physical environments. Our proposed framework incorporates these principles by continuously monitoring components and verifying their integrity (see Section 5.5).

### 3.5. Adversarial Use of AI by Attackers

Perhaps the most concerning emerging threat is the growing capability of attackers to leverage AI technologies against CPS. Advanced AI tools are increasingly being weaponized to discover vulnerabilities, craft sophisticated exploits, and conduct attacks that adapt dynamically to defensive measures. Security experts warn that AI-powered cyberattacks present unique challenges for defenders of critical infrastructure and industrial systems [39]. Unlike traditional attack methodologies with recognizable signatures or patterns, AI driven attacks can continuously evolve their approach, probing for weaknesses and adjusting tactics based on the target’s responses. This creates an asymmetric advantage for attackers, who can potentially deploy automated systems to conduct trial-and-error exploitation at speeds that human defenders cannot match. The threat is significant enough to have attracted governmental attention. The threat has drawn governmental attention; in 2023, U.S. lawmakers formally sought federal assessment of AI-related risks to critical infrastructure and of how defenders might leverage AI in response [40].

Defending against AI-powered threats requires a multi-faceted approach. Organizations must implement robust model designs, enhanced training methods that consider adversarial scenarios, and proactive monitoring systems capable of detecting subtle manipulation attempts. Research suggests that adversarial training—in which models are deliberately exposed to both normal and adversarial inputs during development—can significantly improve resilience against these sophisticated attacks [33].

As this technological arms race accelerates, security professionals must continuously evolve their defensive strategies. This evolution requires ongoing collaboration between academia, industry, and government agencies to share insights on emerging threats and effective countermeasures. Without such cooperation, the gap between offensive and defensive capabilities may widen, potentially leaving critical CPS vulnerable to increasingly sophisticated AI-powered attacks [39]. Just as attackers may harness AI, defenders must do the same. This motivates our framework’s AI-driven approach, aiming to keep defenders a step ahead.

As CPS become increasingly central to our infrastructure, transportation, healthcare, and manufacturing sectors, securing them against these emerging threats becomes not merely a technical challenge but an economic and national security imperative. The future of CPS security will likely involve a combination of advanced AI-powered defenses, regulatory frameworks that address cross-domain risks, and collaborative information sharing between public and private sectors to stay ahead of rapidly evolving threats.

### 3.6. Data Poisoning Attack

In a data poisoning attack, adversaries intentionally manipulate training data to degrade the performance and integrity of ML models—by deleting, modifying, or injecting data so the model produces incorrect outputs. Because CPS uses ML models to predict, control, and optimize physical processes, training on compromised data can impair decision-making and produce unsafe outcomes; for instance, poisoning in smart grids can cause incorrect load forecasting, leading to equipment damage and power outages [41]. Erba et al. [42] demonstrate this in an industrial setting, altering anomalous samples to appear benign in data from a real-time industrial testbed and showing that the resulting poisoning measurably degrades the anomaly-detection system’s performance.

## 4. Leveraging AI Creative Problem Solving Methodologies for Security Assessments

Adapting security assessments to dynamic CPS environments calls for the same ingenuity and adaptability that AI systems exhibit in other complex tasks. We propose to integrate Creative Problem Solving methodologies in AI, namely, problem formulation, knowledge representation, knowledge manipulation (reasoning/search), and solution evaluation into the security assessment lifecycle of CPS. This approach treats the security assessment as an evolving problem that AI can help tackle, rather than a one-time checklist. Below, we outline how each CPS-AI facet can enhance security assessments, and survey state-of-the-art techniques that exemplify these ideas.

### 4.1. Problem Formulation—Dynamic Threat Modeling

The first step is to properly frame the security assessment problem for a given CPS. In AI terms, this is akin to defining the state space, goals, and constraints. Traditional threat modeling (e.g., identifying assets, threats, and attack scenarios) can be augmented with AI to handle the scale and complexity of modern CPS. For example, AI can assist in asset discovery and characterization—machine learning can analyze network traffic and device behavior to automatically identify what assets exist and how they are communicating, a task that is increasingly difficult manually in IoT-rich environments. This adaptive asset identification means the “problem space” of what needs securing is kept up-to-date. Jonathon Gordon notes that AI-driven platforms can rapidly map out CPS assets and their risk profile, answering fundamental questions like “What CPS do I have and how do they connect?” more effectively than manual audits [43]. Once assets and their interdependencies are mapped, AI can help generate threat hypotheses. Knowledge from past incidents and known exploits can be encoded so that the system “imagines” possible attack paths, essentially an AI brainstorming potential threats. This may involve using graph algorithms on a model of the CPS (where nodes are components and edges are connections) to find paths from threat actors to critical assets. It could also involve AI scenario generation, using techniques like combinatorial search or even language model-based simulation to propose “what-if” attack scenarios. Problem formulation in this context sets a moving target: as the CPS evolves (new components, software updates, threat intelligence), the AI continually re-formulates the threat model. This ensures assessments remain relevant and anticipatory.

### 4.2. Knowledge Representation—Digital Twins and Formal Models

Effective security assessment a requires rich representation of both the CPS and attacker knowledge. AI offers tools to represent this knowledge in a machine-interpretable form. One promising approach is the use of digital twins, high-fidelity virtual replicas of CPS, to model system behavior under various conditions. A digital twin can serve as a sandbox for security tests, where AI agents can safely play out attacks without risking the real system [44,45]. By mirroring the physical processes and control logic, the twin provides a knowledge base the AI can manipulate. Recent research shows strong synergy between digital twins and AI for cybersecurity [46]; for instance, AI algorithms can analyze the twin’s telemetry to predict system states and detect anomalies in real time. Beyond simulation, formal knowledge representation is useful, ontologies or knowledge graphs can model CPS components, their trust levels, and known vulnerabilities [47]. An ontology might represent, for example, that “Component X is a camera feeding into AI module Y; if X is compromised, integrity of Y’s decisions is affected.” AI reasoning engines can then infer possible consequences of component compromises. Such representations enable automated reasoning about security, e.g., if a certain sensor is hacked, the system can predict which safety constraints might be violated. Knowledge representation also extends to encoding regulatory requirements, an AI system can have built-in knowledge of standards (like required encryption strength, or data privacy rules) and cross-check the CPS design against these rules, much like a lint tool for compliance. By formalizing both the system model and security policies, AI can automatically flag design elements that deviate from secure practices, which is particularly helpful during development and integration of complex CPS.

### 4.3. Knowledge Manipulation—AI-Driven Analysis and Penetration Testing

Once the problem is defined and knowledge is structured, AI can actively search for vulnerabilities and countermeasures, this is the “knowledge manipulation” or reasoning phase. Techniques here include automated penetration testing, anomaly detection, and what-if analysis, all driven by AI. In recent work, generative AI agents powered by LLMs have been explored as penetration testers [48]. These agents can chain reasoning steps (akin to how a human pentester would) to navigate a complex CPS network, exploit weaknesses, and pivot to deeper access. Gioacchini et al. (2024) introduce AutoPenBench, a framework to evaluate such AI agents, highlighting that while current agents are not yet fully effective (only  21 percent success on complex tasks for a fully autonomous agent), they show promise when combined with human guidance [48]. The ability to automate parts of penetration testing means assessments can be run continuously or on-demand whenever system changes occur, rather than a one-time engagement. Beyond finding exploits, AI can run attack simulations to evaluate system resilience, for example, using reinforcement learning to identify control sequences that could cause unsafe states, which helps in testing CPS for worst-case scenarios. On the defensive side, AI-based Intrusion Detection Systems (IDS) are increasingly deployed in CPS networks. These systems employ machine learning (from simple SVM classifiers to deep neural networks) to learn normal operation patterns and detect deviations indicative of attacks. In an industrial network, an AI IDS might learn the regular modbus command patterns and raise an alert when it sees anomalous sequences that could signify malware controlling a PLC. Because CPS environments often have fairly regular process patterns, anomaly-based detection with AI has proven highly effective [49]; a recent survey catalogs the range of CPS anomaly-detection strategies and their trade-offs [50]. Certain vendors report that AI can understand routine OT device communications and then detect the subtle deviations of a cyberattack, even novel ones. Another manipulation technique is gamification for security training, creating a gamified environment (digital twin) where “blue team” AIs defend and “red team” AIs attack can reveal weaknesses in a controlled setting. Such AI vs. AI simulations help evaluate and improve incident response strategies, essentially stress testing the CPS and its operators. By leveraging AI to manipulate knowledge of the CPS (either searching for flaws or testing defenses), a more thorough assessment is achieved.

### 4.4. Evaluation and Adaptation—Continuous Risk Assessment

The final element is evaluating the findings and iteratively improving security posture. In CPS security, this corresponds to risk analysis and mitigation decisions based on the insights gathered. AI can assist in prioritizing risks by quantitative analysis of potential impacts. For instance, an AI system could simulate the consequences of different attack scenarios (using the digital twin) to estimate physical outcomes like equipment damage or safety hazards, not just data loss. This helps assign more concrete risk metrics (e.g., expected downtime or likelihood of human harm), which align with regulated domain concerns. Moreover, AI can optimize mitigation strategies, given a set of possible countermeasures, an AI planner might suggest the most cost-effective set that maximally reduces risk (solving a kind of optimization problem under constraints like budget or performance impact). Importantly, AI can facilitate continuous monitoring and re-evaluation. This is in line with concepts like the Protective Shell, an autonomous guard that watches the system in real-time. If the IDS or sensors detect an anomaly, AI algorithms quickly evaluate if it is an attack, how severe, and whether to trigger failsafes. In autonomous vehicles, we see early versions of this: some systems use secondary “plausibility checks” (possibly AI-driven) to evaluate decisions of the primary AI, and if something seems off (like the vehicle deciding to accelerate toward an obstacle), an override can occur. In industrial settings, AI-based safety monitors might predict the next state of the physical process and intervene if the predicted state violates safety constraints [4]. From a higher-level perspective, evaluation also means ensuring the security measures themselves do not degrade over time. AI models in security (like detection systems) require periodic retraining with new data to stay effective. Thus, part of the assessment framework is evaluating the AI tools’ performance (false positives, false negatives) and tuning them. Human experts remain in the loop for oversight – as recommended by practitioners, combining AI’s speed with human judgment yields the best results. For example, an AI might flag an unusual pattern; a human analyst verifies if it is a true threat or a benign anomaly, and that feedback is incorporated to refine the model. This continuous loop of assess -> detect -> evaluate -> update embodies the creative problem-solving cycle of treating security as an ongoing puzzle where AI and humans collaborate to adapt to new threats [51].

Integrating AI into CPS security assessments transforms the process from a static compliance exercise into a dynamic, intelligent hunt for vulnerabilities and assurance of safety. By formalizing the assessment problem, representing rich system knowledge, using AI to explore and test, and continuously evaluating outcomes, we achieve a more adaptive and resilient security posture. Next, we consolidate these ideas into a proposed framework and discuss how it aligns with regulatory requirements and can be implemented in practice.

## 5. Proposed AI-Driven CPS Security Assessment Framework

Drawing on the insights above, we propose a conceptual security assessment framework for AI-enabled CPS that interweaves traditional best practices with AI-driven techniques. The framework is designed to be iterative and continuous, reflecting the evolving nature of CPS threats, and it explicitly incorporates Core Problem Solving AI principles (problem formulation, knowledge representation, etc.) at each stage. Crucially, it also aligns with regulatory requirements and standards to ensure that adopting AI-driven assessments strengthens, rather than conflicts with compliance.

We emphasize that the novelty of this framework lies not in any single technique but in their integration into a continuous, standards-aligned assessment loop. Existing approaches address parts of the problem in isolation: standards such as NIST RMF, IEC 62443, and ISO/SAE 21434 provide rigorous but largely periodic, point-in-time assessment; AI-based intrusion and anomaly detection offer continuous monitoring but are typically bolt-on operational tools disconnected from the formal risk-assessment process; and adversarial-ML defenses are studied largely outside any assessment methodology. As summarized in Table 1, none of these simultaneously provides continuous reassessment, full cyber-physical scope, explicit coverage of AI-specific threats, a high degree of automation, and direct mapping to regulatory compliance. Our framework’s contribution is to unify these into a single iterative lifecycle: the threat model is continuously re-formulated as the CPS evolves, digital-twin-based adversarial testing is treated as a first-class assessment activity rather than an afterthought, and compliance traceability is produced as a direct output of each phase rather than reconstructed separately. This integration is what distinguishes the approach from prior work.

**Figure 1:**
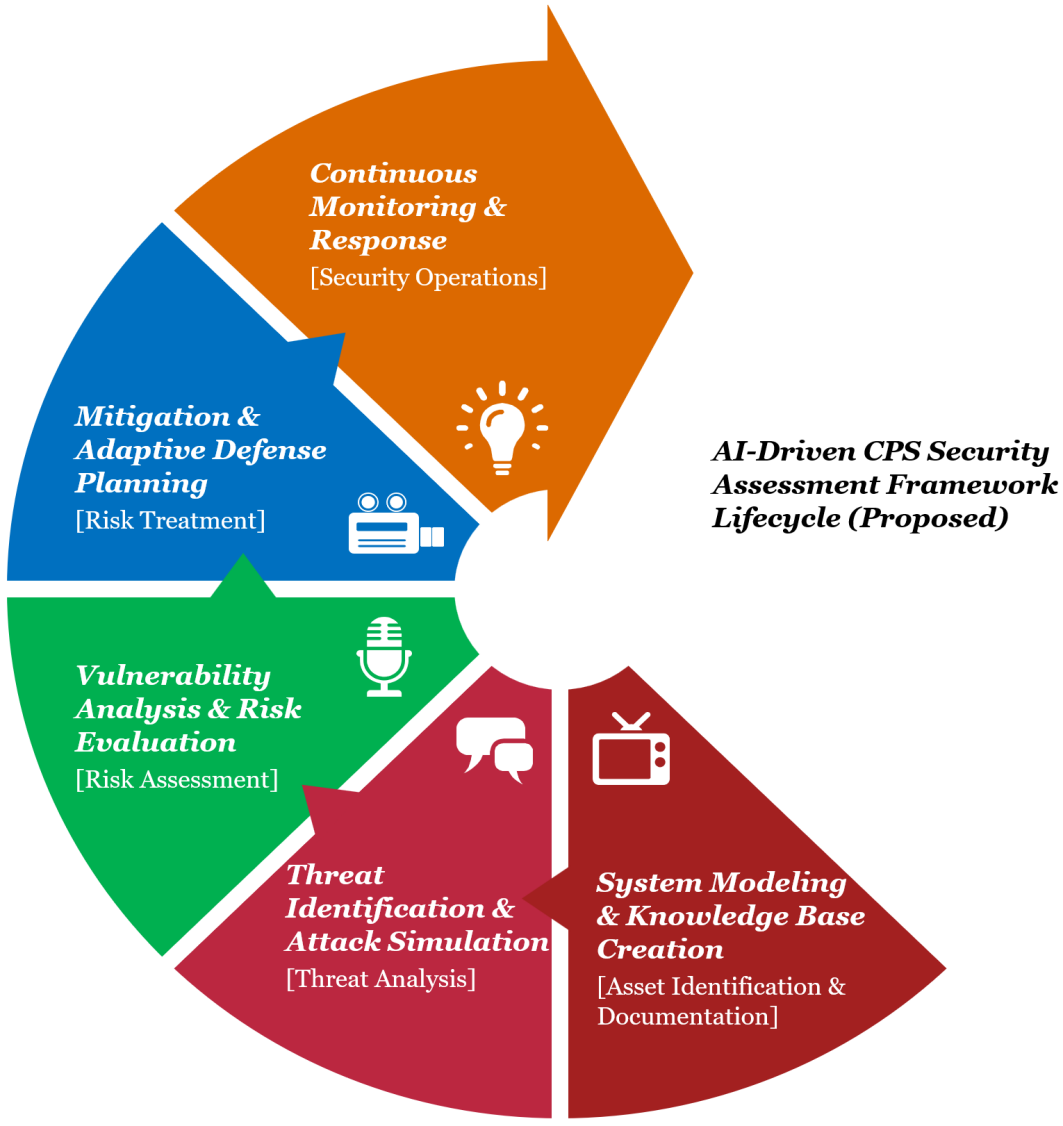
Proposed AI-Driven CPS Security Framework Lifecycle.

Figure 1 illustrates the framework’s phases and their alignment with both AI CPS methodologies and standard security processes. These phases map onto common security lifecycle steps (asset identification, threat analysis, risk assessment, risk treatment, security operations) but are enhanced with AI capabilities.

### 5.1. System Modeling and Knowledge Base Creation

In this initial phase, the CPS is modeled in a unified knowledge base. All assets (physical and digital) are inventoried, and their attributes (function, connectivity, and criticality) are documented. This corresponds to the Identify function of the NIST Cybersecurity Framework and is required by standards like IEC 62443-3-2 [6] (system security requirements and asset identification). We integrate knowledge representation here by building a digital twin or semantic model of the CPS. For example, a power grid CPS model would include substation equipment, sensor telemetry flows, control center links, etc., possibly visualized as a graph. Any AI components (machine learning models, autonomy functions) are highlighted in the model as special assets requiring additional scrutiny (with metadata such as training data source, model type, version). The knowledge base also includes regulatory requirements applicable to each component (e.g., if personal data is processed, GDPR’s data protection requirements are noted) [52]. By structuring this information, we lay the groundwork for automated reasoning. This phase produces a “security digital twin” of the CPS. Regulatory alignment: meets documentation expectations of ISO 21434 (item definition and asset identification in automotive), and GDPR’s record-keeping of data processing activities. It also supports IEC 62443’s requirement [6] to identify zone boundaries and conduits in industrial systems.

### 5.2. Threat Identification and Attack Simulation

Using the model, the framework next performs a comprehensive threat analysis. We leverage AI for problem formulation, the system automatically generates potential threat scenarios against the CPS model. This includes known threat libraries (e.g., known vulnerabilities mapped to components) and AI-generated hypothetical attacks. For each critical asset, AI agents attempt to discover how it could be attacked, for example, trying to identify reachable network paths for a hacker or ways an insider could misuse the system. The generative AI penetration testing approach comes into play here [48]. The framework might launch a suite of simulated attacks in a controlled environment (using the digital twin or a staging system). For instance, it could simulate malware in a PLC to see if the anomaly detection would catch it, or simulate a spoofed sensor feed to test safety responses. Each simulated attack that succeeds (in the twin) is recorded as a potential vulnerability scenario. Importantly, AI helps to prioritize realistic threats, it can incorporate threat intelligence (e.g., current attacker groups tactics) to focus on likely attack vectors. This addresses emergent threats like adversarial ML, the framework would include tests such as attempting to trick vision models with altered images or testing if control AI can be manipulated by abnormal inputs. Human security experts can refine this step by reviewing the AI-generated threats, ensuring plausibility, and adding any scenarios the AI might miss (thus, a human-AI-teamed threat modeling). Regulatory alignment: This phase aligns with requirements for threat assessment in standards (ISO 21434 requires a TARA report, IEC 62443-3-2 requires identifying threat scenarios). The difference is the use of AI to ensure the threat list is exhaustive and up-to-date with emerging tactics. It also follows NIST’s guidance on considering both cyber and physical threats in risk assessments.

### 5.3. Vulnerability Analysis and Risk Evaluation

With a set of possible attack scenarios and system knowledge, the framework evaluates which vulnerabilities exist and what risks they pose. This involves analyzing system configurations, software versions, and even AI model robustness to find weaknesses. Traditional tools (vulnerability scanners, static code analysis) are augmented by AI-driven analytics, e.g., an AI might analyze configuration files and network policies using natural language processing to spot misconfigurations, or use an expert system to correlate multiple low-level findings into a significant vulnerability. Each identified vulnerability is then assessed for impact using the CPS knowledge base. Here, we employ the evaluation aspect of CPS AI in quantifying risk. If an attack can lead to a certain physical outcome (like disabling a safety system), the framework estimates the severity (perhaps using a scale akin to safety integrity levels or the Common Vulnerability Scoring System adapted for physical impact). It also estimates likelihood, potentially using AI to simulate attacker success rates under various conditions, giving a probabilistic risk value. The output of this phase is a prioritized list of risks, each described in terms of scenario, vulnerability, impact (safety, operational, financial, privacy), and compliance implications. For example, a risk might be “Adversarial stop sign attack causes vehicle to miss stops and resulting impact: high safety risk; likelihood: medium; related regulation: violates ISO 26262 [53] road safety requirements if unmitigated.” Regulatory alignment: Risk evaluation results can be directly mapped to compliance reporting. ISO 21434 and IEC 62443 both expect a ranking of risks (often as High/Med/Low) and justification. GDPR would be concerned if any scenario leads to a personal data breach. This phase ensures the framework’s findings are documented in a way that satisfies these requirements (traceability from threat to risk to control measures), which will be used in the next phase.

### 5.4. Mitigation and Adaptive Defense Planning

For each significant risk, the framework recommends security controls or mitigations, essentially building a security improvement plan. AI aids the knowledge manipulation here by searching through a repository of countermeasures (technical fixes, process changes) and even generating novel suggestions. For instance, if a vulnerability is a lack of authentication on a device, the obvious control is to add authentication; if the issue is an AI model susceptible to adversarial inputs, mitigations might include retraining the model with adversarial examples, adding a sensor fusion check, or deploying an input filter. The framework can use multi-objective optimization to pick a set of controls that collectively reduce risk below acceptable thresholds while considering cost and feasibility. We also emphasize adaptive defenses that measures that are dynamic or AI-powered. For example, instead of a static firewall rule, an AI-based firewall could adapt its rules based on detected attack patterns (aligning with the concept of moving target defense). Another example is if supply chain risk for AI models is identified, the mitigation might include instituting a continuous validation program where models are periodically tested for integrity (using techniques like model watermarking or anomaly detection on model outputs). All chosen mitigations are mapped back to the risk scenarios to ensure coverage. This is akin to creating an assurance case where demonstrating that for each identified risk, appropriate controls will be in place. We also ensure compliance mapping, e.g., if the risk was “data privacy breach,” the chosen mitigations would include encryption or access controls that meet GDPR Article 32 (security of processing) requirements [52]. At this stage, it is important to involve human decision makers (engineers, management) to review AI suggestions for practicality and to approve the risk treatment plan, fulfilling governance requirements. Regulatory alignment: This phase aligns with the implementation of security requirements. ISO 21434 [6,7] calls for treatment of risks to meet a tolerable risk level; IEC 62443-3-3 [7] provides a list of security requirements that might be applied. NIST CSF’s Protect and Respond functions are reflected here. Documentation of chosen controls and residual risk is prepared to demonstrate compliance and due diligence.

### 5.5. Continuous Monitoring and Response

Security assessment does not end after deploying controls. The framework establishes a continuous monitoring regimen to validate that controls are effective and to detect new threats. This operational phase integrates AI-driven monitoring as discussed previously about deploying AI-based IDS, anomaly detection on sensor data, health checks for AI components (to catch drift or attacks), and so on. The framework would ingest logs and alerts, and an AI system correlates these to detect potential incidents. If an incident or anomaly is detected, the framework has predefined response playbooks (some possibly automated). For example, if an anomaly suggests a possible Triton-like attack on a safety controller, the protective shell concept might kick in to keep the system safe (e.g., forcing the process to a safe state). The incident is also fed back into the assessment loop: the knowledge base is updated with this new data point, the threat model can be adjusted (perhaps the attacker used a technique not considered before), and the risk evaluation is revisited. This continuous feedback loop ensures the security posture adapts. It also provides assurance to regulators and stakeholders that security is maintained throughout the CPS lifecycle, not just at a single point. Many regulations now emphasize ongoing risk management, for instance, NIST SP 800-37 Risk Management Framework (RMF) [54] requires continuous monitoring as the final step in the cycle. Similarly, automotive regulations (UNECE WP.29) [55] require manufacturers to have cybersecurity monitoring and incident response for vehicles in the field. Our framework’s monitoring phase fulfills these by design. It leverages AI to cope with the volume and velocity of CPS operational data, highlighting critical events for human operators. Over time, metrics from this phase (like number of incidents, time to detect/respond, etc.) can be collected to measure improvement and support the next audit or certification cycle. Table 2 summarizes the implementation mapping by identifying the candidate tools, required inputs, and generated outputs for each phase of the proposed framework.

### 5.6. Human-in-the-Loop and Governance

Throughout the framework, human oversight is maintained to ensure accountability and to address the limitations of AI. Rather than treating human involvement as a general principle, the framework assigns specific roles, responsibilities, and decision authority across its phases, and defines the points at which human approval is mandatory before an automated recommendation may take effect.

Four roles are distinguished. The *security analyst* reviews AI-generated threat scenarios and prioritized risks, validates anomalies raised during monitoring before they are escalated, and filters false positives so that only substantiated findings proceed. The *control or safety engineer* evaluates any mitigation that affects physical operation, confirms the correctness of safe-state transitions and failsafe triggers, and ensures that proposed controls do not compromise process safety or real-time constraints. The *compliance officer* reviews the mapping of controls to regulatory obligations, owns the traceability and audit documentation generated by each phase, and confirms that residual risk is recorded in a form acceptable to auditors. Finally, *management* holds the authority to accept residual risk and to authorize the deployment of countermeasures, fulfilling the governance and accountability expectations of regulated domains.

The framework treats AI outputs as advisory at the points of threat generation, vulnerability analysis, and mitigation suggestion, where automation provides breadth and speed. Two decision gates, however, require explicit human approval. First, no countermeasure that alters physical operation may be deployed without sign-off from the control or safety engineer, ensuring that automated defense does not itself introduce a safety hazard. Second, final acceptance of residual risk rests with management and the compliance officer, and may not be delegated to an automated component. This division preserves the speed of AI-driven assessment while ensuring that consequential, safety- and compliance-critical decisions remain under human control.

To support these decisions, the framework requires that AI components document the evidence underlying their recommendations. When an anomaly is flagged, the supporting sensor readings and log patterns are retained so that an analyst can verify the finding and so that the rationale is available for any subsequent post-incident investigation. The use of explainable AI techniques, where feasible, makes the basis of each recommendation auditable. This approach is consistent with the NIST AI Risk Management Framework [56], whose “Govern” function calls for clearly assigned responsibility, human oversight, and validation of AI outputs. By specifying who is accountable for each decision, at which gate human approval is required, and what evidence must accompany an automated recommendation, the framework embeds governance into the assessment process rather than treating it as an external afterthought.

### 5.7. Evaluation Metrics and Success Indicators

A rigorous assessment framework requires explicit criteria by which its effectiveness can be judged once instantiated. While the present work is conceptual, this section defines the quantitative and qualitative indicators against which the framework is intended to be evaluated in a prototype deployment. The indicators are organized along four dimensions, performance, robustness, adaptability, and compliance, that together capture both the operational efficacy of the framework and its alignment with regulatory expectations. Table 3 summarizes these indicators.

Performance indicators characterize how effectively the framework detects and responds to threats during operation. These include the threat-detection rate (true-positive rate), the false-positive and false-negative rates, the mean time to detect (MTTD) and mean time to respond (MTTR) following the onset of an incident, and the assessment latency incurred per evaluation cycle. Together, these measures quantify both the accuracy and the timeliness of the framework, the latter being critical in cyber-physical settings where delayed detection can translate directly into physical consequences.

Robustness indicators measure the resilience of the framework, and of the AI components it protects, under adversarial conditions. These include the success rate of adversarial-machine-learning perturbations against the protected models and the degradation in detection accuracy observed under data-poisoning of a specified intensity. Because AI-enabled CPS are uniquely exposed to such attacks, robustness is treated as a first-class evaluation dimension rather than a secondary concern.

Adaptability indicators capture the central premise of the framework, namely that security assessment must evolve continuously with the system. These include the time required to re-formulate the threat model following a system or configuration change, the fraction of novel (previously unseen) attacks detected without model retraining, and the recovery time from model drift. These measures distinguish a continuous, adaptive assessment process from periodic, static evaluation.

Compliance indicators relate the framework’s outputs to regulatory obligations. These include the proportion of applicable control requirements covered, mapped to recognized standards such as the NIST Cybersecurity Framework [57], IEC 62443 [6,7], and ISO/SAE 21434 [9]; the completeness of traceability from threat to risk to implemented control; and the audit-readiness of the documentation generated by each phase. These indicators ensure that improvements in security posture can be demonstrated to regulators and auditors, not only to engineers. Table 3 summarizes the evaluation metrics and success indicators used to assess the effectiveness of the proposed framework.

These indicators are not reported with measured values in the present work, as the framework is conceptual; rather, they constitute the evaluation protocol for the prototype deployment identified as the principal direction of future work (Section 7). Establishing baseline values for each indicator, and comparing them against those of conventional periodic assessment, will form the basis of that evaluation.

Here we present (i) dynamic threat-path identification and risk scoring (Phases 5.2 and 5.3), and (ii) continuous anomaly-based monitoring with adaptive response (Section 5.5), including the human-approval gate of Section 5.6.

Algorithm 1 operates on the attributed CPS graph G=(V,E) produced in Section 5.1, where nodes represent assets (sensors, actuators, controllers, AI models, network segments) and edges represent connectivity or trust relationships. Each node carries a criticality weight c(v) and, where applicable, an AI-component flag. Each edge carries an exploitability probability p(e)∈[0,1] estimated from vulnerability data, threat intelligence, or simulated-attack success rate (Section 4.3). Given a set of entry nodes *T* (threat-actor access points) and critical assets *A*, the algorithm searches for attack paths and scores them by combining path-traversal likelihood with the impact of the destination asset, re-running incrementally whenever the graph changes (new asset, patched vulnerability, updated threat intelligence) so that the threat model stays current rather than being recomputed from scratch on a fixed schedule.
**Algorithm 1** Dynamic Threat-Path Identification and Risk Scoring**Require:** 
Attributed CPS graph G=(V,E); entry nodes T⊆V; critical assets A⊆V; edge exploitability p:E→[0,1]; node criticality $c:V\to R_{>0}$; change set ΔG since last run (may be empty)**Ensure:** 
Ranked list *R* of (path, likelihood, impact, risk) tuples  1:$V_{\mathrm{affected}}\leftarrow$ nodes incident to ΔG (or *V* if ΔG=∅, i.e., first run)  2:R←∅  3:**for all** t∈T **do**  4:      L(t)←1.0                                  ▹ cumulative likelihood, initialized at source  5:      Run modified Dijkstra from *t* over *G* using edge weight −logp(e)                             ▹ max-likelihood path search  6:      **for all** a∈A reachable from *t* **do**  7:            **if** path(t,a) intersects $V_{\mathrm{affected}}$ **or** ΔG=∅ **then**  8:                   π← shortest (max-likelihood) path from *t* to *a*  9:                   $L\left(\pi \right)\leftarrow \prod_{e\in \pi }p\left(e\right)$                                        ▹ path likelihood10:                 $I\left(a\right)\leftarrow c\left(a\right)\times ⊮\left[\mathrm{AI}\text{-}\mathrm{component}\;\mathrm{on}\;\pi \right]\times w_{\mathrm{AI}}+c\left(a\right)$            ▹ impact, upweighted if path touches an AI asset11:                 risk(π)←L(π)×I(a)12:                 R←R∪{(π,L(π),I(a),risk(π))}13:           **end if**14:    **end for**15:**end for**16:Sort *R* descending by risk(π)17:**return** *R*

The upweighting term $w_{\mathrm{AI}}$ operationalizes this research’s emphasis on AI-specific threats: paths that traverse an AI-driven component (e.g., a perception model or learned controller) are scored as higher-impact than equivalent paths through conventional components, since a successful compromise there can additionally induce adversarial-input or model-integrity failures beyond a simple loss-of-availability event. Because only $V_{\mathrm{affected}}$ is re-examined on incremental runs, the recomputation cost after a small change (e.g., one new asset or one patched vulnerability) is proportional to the size of the local neighborhood rather than the whole graph, which is what allows Section 5.2’s threat model to be re-formulated continuously rather than only at scheduled audits.

Algorithm 2 formalizes Section 5.5 (continuous monitoring), the “Protective Shell” concept, and the human-approval gate of Section 5.6. It consumes a streaming multivariate telemetry window, scores it with a detector trained on normal operating data, and, upon a sustained anomaly, distinguishes between a bounded automated safe-state response (permitted without a human in the loop, e.g., isolating a segment) and any response that would alter physical operation beyond a safe default (which requires the control/safety-engineer sign-off mandated in Section 5.6 before it may execute).

Persistence-window check (requiring *k* consecutive elevated scores rather than a single spike) is the mechanism used to control the false-positive rate reported in Section 6; larger *k* trades detection latency for a lower false-positive rate, and Section 6 reports this trade-off empirically. Knowledge-base update is what closes the loop between Section 5.5 (monitoring) and Section 5.2 (threat modeling): a confirmed incident becomes a change ΔG that triggers a local re-run of Algorithm 1 rather than sitting in a log until the next scheduled audit.
**Algorithm 2** Continuous AI-Based Anomaly Monitoring and Adaptive Response**Require:** 
Trained detector *D* (fit on normal telemetry); alert threshold τ; persistence window *k*; knowledge base KB; safe-state action set $S_{\mathrm{safe}}$ (pre-approved, does not alter physical operation); restricted action set $S_{\mathrm{restricted}}$ (alters physical operation)**Ensure:** 
Continuously updated alert stream and KB  1:buffer←∅  2:**while** telemetry stream is active **do**  3:      $x_{t}\leftarrow$ next telemetry sample (sensor/actuator readings at time *t*)  4:      $s_{t}\leftarrow -D.\mathrm{score}\left(x_{t}\right)$                                ▹ anomaly score, higher = more anomalous  5:      append $s_{t}$ to buffer; keep last *k* scores  6:      **if** mean(buffer)>τ **then**                         ▹ sustained anomaly, reduces false-positive flicker  7:            evidence← (raw readings, $s_{t}$, buffer history)                         ▹ retained for auditability  8:            analyst ←NotifySecurityAnalyst(evidence)  9:            **if** analyst confirms substantiated anomaly **then**10:                candidate ←SelectResponse(evidence,KB)11:                **if** candidate $\in S_{\mathrm{safe}}$ **then**12:                      execute candidate immediately                           ▹ no physical-operation change13:                **else if** candidate $\in S_{\mathrm{restricted}}$ **then**14:                      **await** control/safety-engineer approval                        ▹ mandatory gate15:                      **if** approved **then**16:                            execute candidate17:                      **else**18:                            fall back to nearest $S_{\mathrm{safe}}$ action (e.g., force safe state)19:                      **end if**20:                **end if**21:                KB←KB∪{evidence,actiontaken}                        ▹ feedback into threat model, triggers Algorithm 122:          **else**23:                label evidence as false positive; use to recalibrate τ24:          **end if**25:      **end if**26:**end while**

## 6. Prototype Implementation and Experimental Evaluation

This section reports an initial empirical evaluation of above-defined algorithms, focused on Algorithm 2 (continuous monitoring) and on the adversarial-robustness. We emphasize a scope limitation up front: the standard public CPS security testbeds used in this literature (e.g., SWaT and WADI from iTrust, Singapore University of Technology and Design, and HAI from the KAIST/dacon consortium) require a data-access request and were not obtainable within the environment used for this evaluation. We therefore built a *synthetic* CPS telemetry testbed, structurally modeled on the sensor/actuator relationships and attack taxonomy used by SWaT-style studies, to obtain a first, reproducible empirical signal for the framework’s monitoring component. This is a proxy evaluation, not a validation on an operational or an established public CPS dataset; obtaining SWaT/WADI/HAI access and repeating this evaluation is identified.

### 6.1. Dataset and Setup

Synthetic CPS telemetry testbed (for Algorithm 2): We simulate a small closed-loop industrial process with six correlated sensors/actuators (tank level, inflow rate, outflow rate, pressure, temperature, valve position), generated as a quasi-periodic base signal plus first-order autoregressive noise, so that sensors exhibit the realistic cross-correlation that a physical process induces (e.g., tank level tracks the inflow/outflow balance). A training stream of 20,000 timesteps of normal operation is generated, followed by an 8000-timestep test stream into which 9–10 attack windows (length 80–300 timesteps each) are injected at random offsets, using four attack types drawn directly from the taxonomy of defined vulnerabilities: *spoofing* (replacing a sensor’s true value with an attacker-controlled value), *freezing* (replaying a stale value, e.g., to hide a physical change from the operator, as in the Stuxnet centrifuge case of Section 4.2), *ramping* (a slow linear drift, representative of a stealthy manipulation), and *flooding* (injected high-variance noise, representative of sensor-jamming or DoS-style interference). The detector (Algorithm 2’s *D*) is an Isolation Forest trained only on the normal-operation stream, and the alert threshold τ is calibrated as the 98th percentile of anomaly scores on held-out normal data, following the calibration step implicit in Algorithm 2.

Adversarial-robustness benchmark: As a proxy for the vision-based perception attacks discussed in Section 3.1 (sticker-based stop-sign misclassification), we train a linear softmax classifier from scratch (so that exact gradients are available) on the scikit-learn digits dataset (1797 8×8 grayscale images, 10 classes), a small, locally available image-classification task standing in for a gauge- or sign-reading vision module. We use a 75/25 train/test split and apply the Fast Gradient Sign Method (FGSM) at perturbation budgets ϵ∈{0,0.02,0.05,0.1,0.2,0.3} (pixel values normalized to [0,1]), measuring accuracy under attack and the fraction of originally-correct test samples flipped to an incorrect label. This experiment is a controlled proxy, not a reproduction of the physical sticker-attack literature; its purpose is to instantiate the Robustness metric with a genuine, reproducible measurement rather than leaving it undefined. All experiments are fully reproducible: fixed random seeds are used throughout, and both scripts run in under one second on a standard CPU with only NumPy and scikit-learn.

### 6.2. Anomaly Detection Results

Table 4 reports the measured results of Algorithm 2 on the synthetic testbed described above.

Every injected attack window was detected at least once, and the detector flagged the average attack within 19 timesteps of onset, i.e., the framework’s continuous-monitoring loop (Section 5.5, Algorithm 2) surfaces an alert well before an attack window (minimum length 80 timesteps) has run its course, at a modest 2.3% false-positive rate on normal data. The gap between the per-attack detection rate (100%) and the point-wise recall (22.4%) is expected and informative: the detector reliably *notices* each attack shortly after onset but does not flag every timestep within a slow-drifting attack window (particularly the *ramp* attacks, whose early timesteps are close to normal operating range by construction). This matches the persistence-window design of Algorithm 2 (line 6), which is intended to catch sustained deviation quickly rather than to score every single timestep as anomalous, and it argues for reporting event-level detection rate and MTTD—not point-wise recall alone—as the primary operational metrics, consistent with how frames Performance around MTTD/MTTR rather than pure classification accuracy.

### 6.3. Adversarial Robustness Results

Table 5 reports the measured FGSM robustness sweep. The clean-data test accuracy of the proxy perception model is 94.9%.

Even small, human-imperceptible perturbation budgets produce measurable degradation (ϵ=0.02 already costs 2.7 accuracy points), and degradation accelerates sharply beyond ϵ=0.1, where more than a quarter of previously correct classifications flip. This mirrors, at small scale, the practical concern documented in Section 3.1 for physical sticker attacks: a perception component with no adversarial-robustness treatment can be pushed to near-total failure well within a perturbation budget that would be visually subtle in a physical analog. The result is reported here specifically to give the Robustness dimension, non-zero baseline against which a mitigated version of the same model (e.g., trained with adversarial examples, per Section 4.4) could be compared in future work; we did not implement adversarial training in this evaluation, so the numbers above should be read as an *unmitigated baseline*, not a statement about the framework’s mitigation efficacy.

The Performance and Robustness rows now have measured values from Section 6.2 and Section 6.3 (synthetic-proxy setting); the Adaptability and Compliance rows remain defined but unmeasured, pending the full prototype deployment.

## 7. Case Study: Securing a Smart Manufacturing Plant from a Ransomware Attack

**Figure 2:**
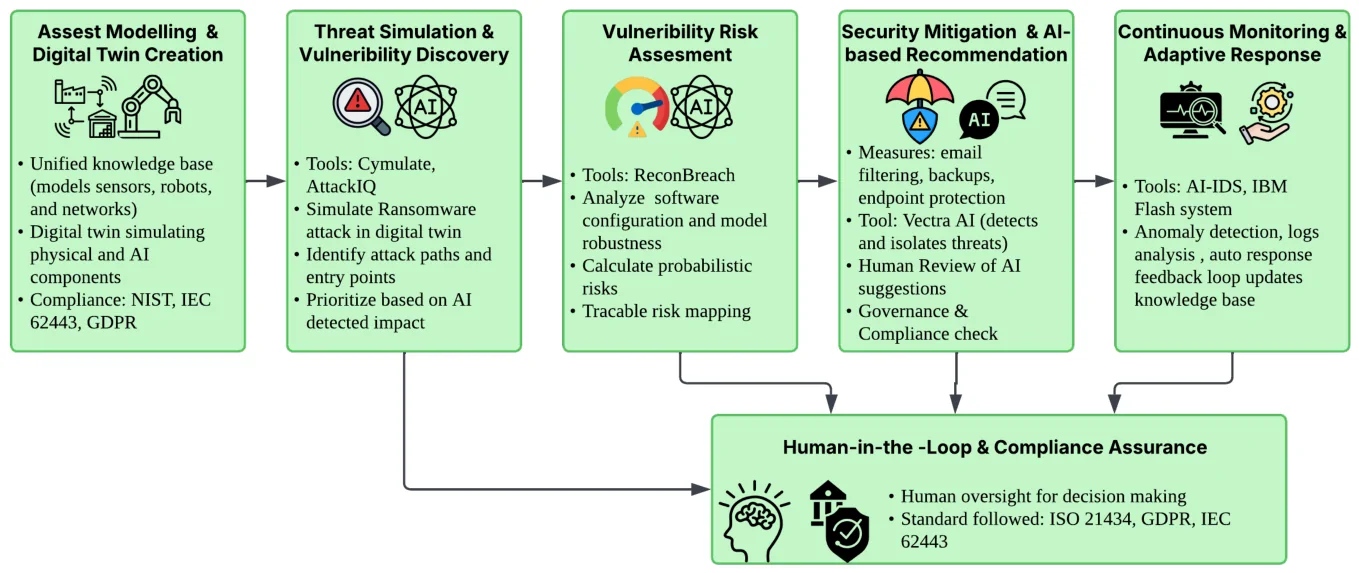
Case study of securing a smart manufacturing system against ransomware attacks using the proposed framework.

A smart manufacturing plant comprises several industrial robots and sensors controlled by control systems, shown in Figure 2. This intelligent system can be targeted by a ransomware attack where the attacker tries to encrypt the critical system information and demand a ransom to reinstate the system’s regular operation, resulting in the system’s downtime and significant financial loss. The following paragraphs explain how our proposed systems deal with this attack scenario.

The framework is designed to integrate with the operational infrastructure already present in industrial cyber-physical environments rather than to replace it. In the system-modeling phase, asset, topology, and process data are ingested from supervisory control and data acquisition (SCADA) systems, process historians, and industrial Internet-of-Things (IIoT) platforms to populate the knowledge base, so that the model reflects the deployed system rather than an idealized design. The digital twin is constructed using established twin frameworks and is synchronized with live operational telemetry, allowing threat simulation and risk evaluation to be carried out against an accurate, continuously updated replica without disturbing the production system. In the monitoring phase, runtime data are consumed through standard industrial communication protocols such as Modbus and OPC-UA and forwarded to AI-based intrusion-detection and anomaly-detection components, such as those described in the case study (Section 6). Detections are fed back into the knowledge base, closing the loop between operational monitoring and the assessment process. This integration pattern allows the framework to be adopted incrementally, attaching to existing SCADA, digital-twin, and IIoT deployments, rather than requiring a wholesale replacement of plant infrastructure.

As discussed in the first stage of the proposed AI-driven CPS security framework lifecycle, all the physical and digital assets of smart manufacturing systems, including sensors, industrial robots, communication networks, and control systems, are modeled in the unified knowledge base. We then create digital twins of the manufacturing plant that mimic all its components and their interactions. The digital twin must also showcase all AI components, including ML models utilized for predictive maintenance. The regulatory requirements should guide the execution of all these tasks. For example, the modeled knowledge base should comply with the NIST cybersecurity framework, IEC 62443, and GDPR to protect crucial information.

In the next phase, we utilize AI-based tools like Cymulate and AttackIQ to identify potential threats to the manufacturing systems [58]. These tools are capable of generating known attacks and hypothetical vulnerabilities. For instance, Cymulate can simulate ransom attacks by mimicking the techniques and processes of the real-world adversary. Furthermore, AI tools can explore how the critical elements of the systems can be compromised; for example, figuring out the path in the communication network from where a hacker enters the system. In the controlled environment of a developed digital twin, we simulate the ransomware attack and record each successful simulated attack, which is a potential vulnerability. AI-based tools help us prioritize the most realistic threats and focus on likely attack vulnerabilities.

Next, we scrutinize the software versions, system configuration, and the resilience of AI models to pinpoint vulnerabilities using AI-enabled tools. Then, we calculate the probabilistic risk value by assessing impact and likelihood with the AI-simulated attack success rate for each vulnerability. Thereafter, we get the lists of prioritized risks based on their scenario, vulnerability, and impact on safety, operation, finance, and privacy that trace from threat to risk to control measures.

In the fourth phase, we recommend different security measures to mitigate the attack, such as enhancing email filtering to combat phishing, maintaining robust backup solutions, and deploying endpoint protection systems to block ransomware. Modern AI-based tools like Vectra AI [59] can adapt to identify attack patterns dynamically and recommend the best solutions. For example, Vectra AI can identify recent ransomware activities and automatically separate the affected components in the system to ensure the attack does not spread further to other elements. Then, the security personnel review the suggestions provided by these AI tools and determine the practicability, feasibility, and effectiveness of these recommended tools, keeping in mind the governance compliance and the regulatory standards.

After mitigation and adaptive defense planning, the next stage is continuously monitoring and responding to threats. We use AI-driven IDS, anomaly detection, and health checks for AI components to monitor the system continuously. IBM FlashSystem is an example of an ML-based tool that can monitor the pattern of data and detect anomalies indicating ransomware in a very short period of less than 60 s. AI analyzes logs and alerts, correlating the data to identify potential incidents. Protection techniques activate the predefined solutions upon detecting anomalies by pushing the process to a safe state if necessary. The future incident data is fed back into the assessment loop, thereby updating the knowledge base to make sure that the security mechanisms are robust over time and become proactive. We maintain human supervision throughout the process to overcome the limitations of AI and ensure accountability. Human experts finalize the key decisions, such as deploying solutions and accepting the final risk. The AI tools utilized keep a record of proof of their recommendations, contributing to the transparency and auditability of the procedures. The framework ensures all actions follow established security standards like ISO 21434, IEC 62443, and GDPR. Combining strict compliance requirements with the flexibility of AI helps build a strong and proactive security system for the smart manufacturing plant.

We present this case study as an illustrative walkthrough of how the framework’s phases would operate against a concrete threat, rather than as an empirical evaluation. Its purpose is to demonstrate conceptual coverage and internal consistency; quantitative validation on a deployed or emulated testbed is left to future work (Section 7).

## 8. Conclusions and Future Work

Security assessment for AI-driven CPS in regulated domains must evolve beyond traditional checklists and one-off evaluations. In this study, we reviewed the landscape of CPS security methodologies across industries and found that while standards and frameworks provide a necessary foundation, they have notable gaps when confronted with emerging digital-physical threats and AI-related vulnerabilities. We discussed how autonomous and intelligent CPS introduce new attack surfaces from adversarial sensor inputs that can deceive AI to malware that can disable physical safety mechanisms, which demand more adaptive and continuous security strategies.

To address these challenges, we explored the fusion of AI Creative Problem Solving techniques with CPS security assessment. By treating the assessment process itself as an AI problem, involving automated threat modeling, knowledge-rich system representations, AI-driven penetration testing, and continuous learning, defenders can stay one step ahead of evolving threats. We proposed a conceptual framework that operationalizes this approach, integrating dynamic risk assessment tools (like AI-powered anomaly detection and automated attack simulation) into the CPS lifecycle. This framework emphasizes resilience and adaptability, enabling the system to monitor its own security posture at runtime and respond to incidents in real-time, complementing design-time analysis. We also ensured the framework is grounded in regulatory compliance, mapping AI-enhanced practices to existing standards (NIST, IEC 62443, ISO 21434, GDPR) so that organizations can innovate in security without running afoul of requirements.

We acknowledge that this work is conceptual: the framework has not yet been implemented end-to-end or evaluated empirically, and the case study is illustrative rather than experimental. The constituent techniques are individually validated in the literature, but their integrated effectiveness—including overhead, false-positive rates, and the practical cost of maintaining the digital twin and knowledge base—remains to be measured. Establishing these metrics through a prototype deployment is the primary objective of our future work.

In conclusion, our research contributes a timely and comprehensive perspective on securing next-generation CPS. The integration of AI into both the operation and protection of CPS is inevitable; harnessing AI for defense is essential to avoid falling behind attackers who will undoubtedly do the same. The proposed methodology enables a shift from reactive, compliance-driven security to proactive, intelligence-driven security. We anticipate that as AI continues to mature, tools like autonomous penetration testers, digital twin simulations, and predictive risk analytics will become staples of CPS security programs. This work lays a conceptual foundation for that future and invites further development and validation. Future work will involve prototyping elements of the framework in specific domains (e.g., deploying a pilot “protective shell” in a smart factory environment) and rigorously evaluating the outcomes, measuring improvements in threat detection rates, reduction in vulnerabilities over time, and compliance efficiency. We also recognize the importance of addressing potential downsides of AI (such as false positives or the need for transparency), which will be an ongoing consideration.

Ultimately, by combining the strengths of human expertise, established security practices, and AI, we can achieve comprehensive, continuous, and compliant CPS security assessments. This aims to foster safer and more secure CPS in an era when they are increasingly autonomous and interconnected. The insights from this research can inform both practitioners looking to enhance their security toolkit and researchers seeking to innovate at the intersection of AI and CPS security. We hope this study stimulates further discussion and collaboration on building AI-empowered security assessment frameworks that keep our critical systems a step ahead of adversaries.

## Figures and Tables

**Table 2:** Implementation mapping: candidate tools, inputs, and outputs per framework phase.

Phase	Candidate Tools/Methods	Inputs	Outputs
System Modeling & KB (5.1)	Digital-twin platforms; ontology/knowledge-graph tools	Asset inventory, network topology, regulatory rules	Attributed CPS graph *G*; security digital twin
Threat ID & Simulation (5.2)	Cymulate, AttackIQ; LLM-based pentest agents	Graph *G*; threat-intel feeds	Ranked attack paths; simulated-attack log
Vulnerability & Risk Eval (5.3)	ReconBreach; vulnerability scanners; NLP config analysis	Attack paths; configs, software versions	Prioritized risk list (impact, likelihood)
Mitigation Planning (5.4)	Multi-objective optimizers; Vectra AI	Risk list; control repository; cost constraints	Risk-treatment plan; control–risk mapping
Continuous Monitoring (5.5)	AI-based IDS; IBM FlashSystem; anomaly detection	Runtime logs, sensor telemetry	Alerts; safe-state triggers; KB updates

**Table 3:** Evaluation metrics and success indicators for the proposed framework.

Dimension	Metric	Definition/Direction
Performance	Threat-detection rate; false-positive and false-negative rates; MTTD; MTTR; per-cycle assessment latency	Accuracy and timeliness of detection and response; higher detection rate and lower error rates, MTTD, MTTR, and latency are preferred
Robustness	Adversarial-perturbation success rate; accuracy degradation under data poisoning	Resilience of protected AI models under adversarial inputs; lower values are preferred
Adaptability	Threat-model re-formulation time; fraction of novel attacks detected without retraining; model-drift recovery time	Capacity to track an evolving system; higher novel-detection fraction and lower re-formulation and recovery times are preferred
Compliance	Control-requirement coverage (NIST, IEC 62443, ISO/SAE 21434); threat-to-control traceability completeness; documentation audit-readiness	Alignment with regulatory obligations; higher coverage, completeness, and audit-readiness are preferred

**Table 4:** Measured anomaly-detection results on the synthetic CPS telemetry testbed (single run, seed = 42).

Metric	Measured Value
Attack windows injected	9
Attack windows detected (per-attack, at least one alert in window)	9 (100%)
Point-wise precision	0.729
Point-wise recall	0.224
Point-wise F1	0.342
False-positive rate (normal timesteps flagged)	2.34%
Mean Time To Detect (timesteps after attack onset)	19.2

**Table 5:** Measured adversarial-robustness sweep (FGSM, proxy vision classifier, seed = 0).

ϵ	Accuracy Under Attack	Accuracy Degradation	Attack Success Rate
0.00	94.89%	–	–
0.02	92.22%	2.67 pts	2.81%
0.05	85.78%	9.11 pts	9.60%
0.10	68.44%	26.44 pts	27.87%
0.20	19.11%	75.78 pts	79.86%
0.30	0.22%	94.67 pts	99.77%

## Data Availability

The data generated during this study are not publicly available, as they have not been prepared for public release. This research does not involve dual-use research of concern (DURC) as defined by applicable national or international guidelines. The study focuses on the assessment of AI-driven problem-solving approaches for cybersecurity in cyber-physical systems and does not involve biological agents, pathogens, or research activities that pose a significant risk to public health or national security.
